# Neuroprotection with rasagiline in patients with macula-off retinal detachment: A randomized controlled pilot study

**DOI:** 10.1038/s41598-020-61835-0

**Published:** 2020-03-18

**Authors:** Siqing Yu, Carsten Framme, Marcel Nico Menke, Lieselotte Erika Berger, Martin Sebastian Zinkernagel, Marion Rohit Munk, Sebastian Wolf, Andreas Ebneter

**Affiliations:** 1 Department of Ophthalmology and Department of Clinical Research, Inselspital, Bern University Hospital, University of Bern, Bern, Switzerland; 2 Klinik für Augenheilkunde, Medizinische Hochschule Hannover, Carl-Neuberg-Str. 1, Hannover, D-30625 Germany; 3 Department of Ophthalmology, Kantonsspital Aarau, Herzogstrasse 15, Aarau, Switzerland

**Keywords:** Drug discovery, Retinal diseases

## Abstract

We aimed to evaluate the neuroprotective efficacy of rasagiline in pseudophakic patients who had surgery for macula-off rhegmatogenous retinal detachment (RRD). This was a 6-month, prospective, randomized, double-blind, placebo-controlled pilot study. Patients presenting with acute macula-off RRD were recruited and randomized 1:1 to receive rasagiline 1 mg/day or placebo for 7 days. Best-corrected visual acuity (BCVA) and optical coherence tomography were acquired 1 day before as well as 2 days, 3 weeks, 3 months and 6 months after surgery. We screened 26 patients with RRD whereof 23 were eventually included and randomized. The primary outcome was final BCVA. Secondary outcomes included central retinal thickness (CRT) and adverse events (AE). We evaluated photoreceptor cells (prc) recovery through morphological measurements. The baseline characteristics were comparable between groups. BCVA significantly improved in both groups (letters gained: rasagiline 61.5 ± 18.1 vs placebo 55.3 ± 29.2, p = 0.56), but no significant inter-group difference was found at any visit. CRT was stable 3 weeks after surgery onwards, with no inter-group difference. No treatment-emergent AE occurred. Significant prc restoration was observed from 3 weeks to 6 months after surgery, without inter-group difference at either visit. Ellipsoid zone integrity (β = 0.517, p = 0.008) and foveal bulge (β = 0.387, p = 0.038) were significant predictors of good final BCVA. In conclusion, perioperative oral treatment with rasagiline 1 mg/day for 7 days did not show significant benefits on visual or anatomical outcomes in macula-off RRD patients.

## Introduction

Rhegmatogenous retinal detachment (RRD) refers to separation of the neurosensory retina from the retinal pigment epithelium (RPE) due to accumulation of subretinal fluid through one or more retinal breaks^1^. Currently, surgical repair is the only available treatment. Foveal detachment (macula-off) occurs in 54.5% of all RRD patients and is a major factor influencing visual recovery after successful surgical repair^2^.

As a result of deprived metabolic supply from the RPE and choroidal vasculature, photoreceptor (prc) death is the ultimate cause of vision loss after RRD^3^. This is particularly relevant in the macula, where the density of prc is the highest and maximal visual acuity is achieved. Arguably, visual acuity recovery is significantly limited due to prc loss. Apoptosis was believed to be the major cause involved in prc death. However, recent evidence demonstrated that non-apoptotic forms of cell death (autophagy and necrosis), mitochondrial outer membrane permeabilization and inflammation raised by the interaction between dying cells and phagocytes all play a role in prc death after RRD^4^. Accordingly, strategies targeting these pathways may be effective in prc rescue after RRD.

Rasagiline [N-propargyl-1-(R)-aminoindan] is a second-generation propargylamine pharmacophore that selectively and irreversibly inhibits monoamine oxidase B (MAO-B). It has been an approved drug for the treatment of Parkinson’s disease since the 2000s and mitigates oxidative stress in the brain^5^. Moreover, rasagiline had neuroprotective potential in preclinical studies that showed that the N-propargyl moiety promotes mitochondrial viability and stabilizes permeability transition by regulating Bcl-2 family proteins^6^.

These findings prompted exploration of the neuroprotective effect of rasagiline in retinal disease. One experimental study assessed prc neuroprotection by rasagiline in Prph2/rds mice, a well-characterized mouse model of retinal degeneration. Eigeldinger-Berthou *et al*. demonstrated that rasagiline not only delayed the activation of effector caspases and the subsequent downstream apoptosis, but also affected autophagy induction and inflammation activity^7^. Recently, Garcia-Delgado *et al*. showed rasagiline delayed prc degeneration via modulation of Bax/Bcl-2 expression in rd10 mice^8^. Besides, rasagiline enhanced survival of retinal ganglion cells in an experimental glaucoma model^9^ and in a retinal ischemia-reperfusion injury model when it was combined with idebenone^10^. However, no clinical studies have yet investigated the effect of rasageline in patients with RRD.

The standard 1 mg dose of rasagiline in treatment for Parkinson disease has a high safety profile^11^. The bioavailability of rasagiline from oral administration was reported to be sufficiently concentrated in the retina in mice^7^. Here, we present the first clinical trial investigating the neuroprotective effects of perioperative oral rasagiline (1 mg daily for 7 days) in pseudophakic patients with macula-off RRD.

## Methods

### Study design and participants

This was a single-center, randomized, double-blind, placebo-controlled trial evaluating the neuroprotective effect of rasagiline in patients with macula-off RRD. This study was conducted between September 2010 and December 2018 at the Department of Ophthalmology, Bern University Hospital, Switzerland. The trial was approved by the local ethics committee, Kantonale Ethikkommission (KEK) Bern (approved number, KEK 178/10) as well as the regulatory authority Swissmedic, and registered at ClinicalTrials.gov (NCT02068625). The study was conducted in accordance with the Declaration of Helsinki. Patients had to provide written informed consent before any study specific procedures.

Included participants were adult pseudophakic patients suffering from macula-off RRD with reported central vision affection of less than 72 hours at presentation. Exclusion criteria were participation in other clinical trials, narrow angle glaucoma, previous intraocular surgery other than cataract operation, any other retinal disease such as age-related macular degeneration, concurrent treatment with MAO inhibitors (selective or non-selective), pethidine, fluoxetine or fluvoxamine in last 14 days, pregnancy or lactation, malignant arterial hypertension (diastolic pressure over 105 mmHg), liver or kidney failure, and any other proximately life-threatening or malignant disease.

### Randomization and masking

Randomization took place after informed consent and confirmation that inclusion criteria were met. The trial was double-blinded. The study medication, i.e., placebo or rasagiline (Teva Pharmaceutical Industries Ltd, Netanya, Israel) was prepared by the Inselspital hospital pharmacy in identical pills and stored in neutral vials.

### Procedures

Study participants received perioperative oral treatment with either rasagiline (1 mg) or placebo once daily for 7 days, which was initiated at the time of hospital admission (baseline visit), usually one day before planned surgery the next day. Standard 3-port pars plana vitrectomy was performed with 20- or 23-gauge instruments, and sometimes an encircling band was added for external support. Hence, the specific choice of the detailed surgical procedure was at the discretion of the operating surgeons (S.W., M.S.Z., M.N.M., A.E.). Four follow-up visits took place at 2 days, 3 weeks (±5 days), 3 months (±10 days) and 6 months (±10 days) after the surgery. At each visit, a thorough ophthalmic exam was completed, including best-corrected visual acuity (BCVA) assessed with the Early Treatment Diabetic Retinopathy Study (ETDRS) chart, slit-lamp Goldman applanation tonometry, pupil dilation, color fundus photographs including autofluorescence, and spectral-domain Optical Coherence Tomography (SD-OCT; Spectralis, Heidelberg Engineering, Germany). Investigators questioned patients about medication change or intercurrent adverse events (AE) and instructed patients to report any such future events.

### Outcomes

The primary outcome was BCVA 6 months after surgical repair. Secondary efficacy outcomes were central retinal thickness (CRT) at month 6, measured in the ETDRS grid’s central subfield. Safety endpoints included the type and frequency of reported AEs and serious AEs. Additionally, thickness of four different layers at week 3 and month 6 (visits 2 and 4) was measured using a commercial software (Orion, Voxeleron LLC, San Francisco, USA): ganglion cell complex (GCC), inner nuclear layer-outer plexiform layer complex (INL-OPL), outer nuclear layer (ONL) and external limiting membrane (ELM)-RPE. To evaluate prc recovery, we graded the integrity of the outer retinal bands, namely ELM, ellipsoid zone (EZ) and cone interdigitation zone (CIZ)^12^. The integrity was graded at the fovea in a 1-mm-diameter area on a 5-point scale as follows: (1) line not visible; (2) line disruption >500 μm; (3) line disruption >200 μm, but <500 μm; (4) line disruption <200 μm; (5) continuous line. Lastly, we graded the presence of the foveal bulge at the end of the study, an indicator of good foveal microstructure. A foveal bulge was defined as an EZ-RPE thickness at the central fovea >10 μm greater than the average EZ-RPE thickness at 250 μm temporal and nasal to the central fovea^13^.

### Statistical analysis

Surgical success and failure rates were calculated. The failure rate was expressed in two ways^14^. The primary failure rate was the percentage of eyes that had a recurrent detachment or a complication after the initial procedure requiring an additional surgery but no silicone oil. There was a second group of patients who developed severe proliferative vitreoretinopathy (PVR) that was managed with silicone oil tamponade left in place for at least six months. These eyes had silicone oil remaining at the end of the study and their final visual outcomes pending. These eyes were not included in outcome analysis.

**Figure 1:**
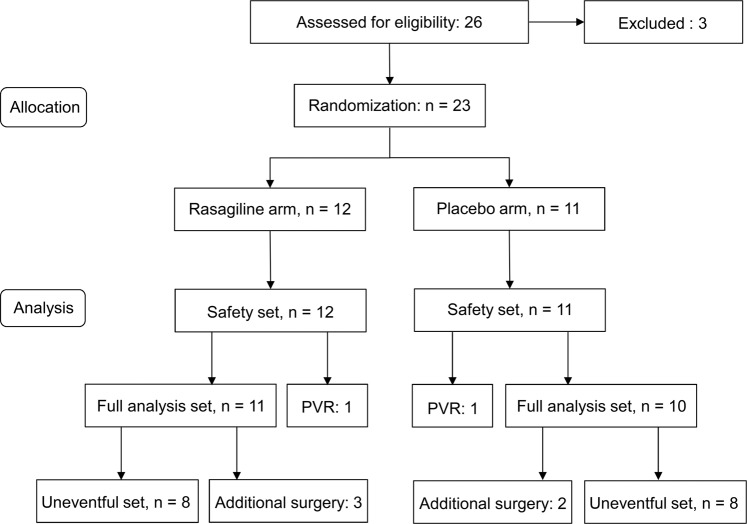
Flow diagram of the trial.

Analyses were performed on three sets. The safety set consisted of all randomized participants who took the perioperative oral medication for 7 days and underwent at least 1 safety assessment after enrolment. The full analysis set (FAS) consisted of all participants who completed the trial and did not develop PVR managed with silicone oil tamponade and had reliable visual acuity data at study end 6 months after initial repair. The uneventful set consisted of all randomized participants completing the study without the need for any additional intraocular surgery to address complications (Fig. 1).

Continuous data were summarized using mean and standard deviation. We used Student’s t-test to test the null hypothesis of no difference between the means of the two randomized groups. Categorical data were summarized using percentages, and the groups were compared using Fisher’s exact test. We constructed linear regression models to evaluate the effect of variables on study outcomes. Statistical testing was two-sided with a 5% significance level.

## Results

### Baseline characteristics

A total of 26 participants signed informed consent and entered screening. Three participants did not meet inclusion criteria. Hence, the safety set included 23 patients (rasagiline, n = 12; placebo, n = 11).

Baseline characteristics were well balanced for demographic parameters, BCVA, and duration of symptoms before presentation group (Table 1). The average age was 68.0 ± 10.5 years, 78% were male. There was no significant difference between groups for any of the parameters (p > 0.05).

**Table 1:** Baseline characteristics of the participants in the safety set.

	Rasagiline (n = 12)	Placebo (n = 11)	Total (n = 23)
Age (yrs), mean (SD)	67.2 (9.7)	69.0 (11.7)	68.0 (10.5)
Gender			
Female	3 (25%)	2 (18%)	5 (22%)
Male	9 (75%)	9 (82%)	5 (78%)
Study eye			
Right	6 (50%)	6 (55%)	12 (52%)
Left	6 (50%)	5 (45%)	11 (48%)
BCVA			
ETDRS letters, mean (SD)	14.3 (20.2)	19.5 (27.1)	16.8 (23.4)
≥30 letters	2 (17%)	4 (36%)	6 (26%)
IOP (mmHg), mean (SD)	13.1 (3.4)	13.2 (4.0)	13.1 (3.6)
Duration vision loss before presentation			
≤24 hours	3 (25%)	1 (9%)	4 (17%)
24–72 hours	9 (75%)	10 (91%)	19 (83%)
Current treatment with ASA	4 (33%)	4 (36%)	8 (35%)
Height of macular detachment (μm)	707.0 (307.8)	771.3 (364.8)	739.2 (329.1)

Height of macular detachment were gradable in 18 participants (9 in each group) in the safety set.

BCVA = best-corrected visual acuity, ETDRS = Early Treatment Diabetic Retinopathy Study, IOP = intraocular pressure, ASA = acetylsalicylic acid.

### Surgical success rate

SF6 gas tamponade was used in all 23 primary surgeries. In total, 16 participants were successfully repaired by single surgery, achieving a single operation success rate (SOSR) of 70% (rasagiline, 67%; placebo, 73%). Five recurrent detachments (rasagiline, n = 3; placebo, n = 2), one full thickness macular hole (FTMH; rasagiline group), and one severe epiretinal membrane (ERM; placebo group) occurred during the study period, leading to a primary failure rate of 30%. Two of the re-detachments, one in each arm, received two additional surgeries and were excluded from the FAS due to remaining silicone oil and severe PVR at the end of study. Thus, the PVR rate was 9%. The other cases were managed by one additional surgery. Therefore, 21 participants (91%) completed the trial and were included in the FAS (rasagiline, n = 11; placebo, n = 10). 16 participants, 8 in each arm had an uncomplicated postoperative course, forming the uneventful set. Details of the additional surgeries during the study follow-up are listed in Table 2.

**Table 2:** Additional surgery requirement of the participants in the safety set.

	Rasagiline (n = 12)	Placebo (n = 11)	P value
Unsuccessful repair	4 (33%)	2 (18%)	0.64
Recurrence of retinal detachment	3 (25%)	2 (18%)	1
Time to first recurrence (days), mean (range)	23.0 (18–29)	29.5 (12–47)	0.67
Number of additional surgery, mean (range)	1.3 (1–2)	1.5 (1–2)	0.79
Silicone oil endotamponade	2 (67%)	2 (100%)	1
Proliferative vitreoretinopathy development	1 (33%)	1 (50%)	1
Full thickness macular hole	1 (8%)	—	—
Time to additional surgery (days)	41	—	—
Epiretinal membrane	—	1 (9%)	—
Time to the additional surgery (days)	—	73	—

### Visual outcome

**Figure 2:**
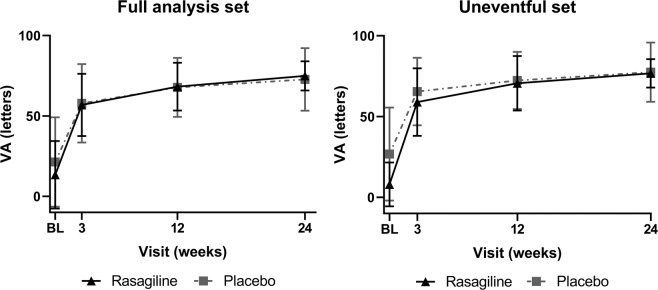
Mean BCVA change during the follow-up of the participants in the FAS and the uneventful set. Mean BCVA significantly increased during the observation period in both groups of both sets. Large improvement happened during the first 3 weeks. There was no difference at any visit between the rasagiline and placebo groups in BCVA (P > 0.05). BL = baseline.

BCVA significantly improved in both groups after surgery (letters gained in 6 months, FAS: 61.5 ± 18.1 in rasagiline and 55.3 ± 29.2 in placebo group; Uneventful set: 68.8 ± 9.1 in rasagiline and 50.8 ± 30.6 in placebo group), but there was no significant difference of final BCVA between groups (Rasagiline 74.9 ± 9.1 letters vs. placebo 72.7 ± 19.5 letters, Table 3). The most significant improvement in BCVA was noted during the first three weeks after the surgery (FAS: 43 letters in rasagiline and 36 letters in placebo group; Uneventful set: 51 letters in rasagiline and 39 letters in placebo group), with no significant difference between groups. There was no difference at any visit between the rasagiline and placebo groups in BCVA (Fig. 2). Final BCVA of the two PVR cases with silicone oil tamponade *in situ* were finger counting and 20 letters, respectively. The other eyes requiring additional surgery reached a mean final BCVA of 63.8 letters (range: 45–81), and 75% of the eyes had 60 ETDRS letters or better BCVA.

**Table 3:** Final visual acuity at month 6 of the participants in FAS and uneventful set.

	Full analysis set			Uneventful set		
	Rasagiline (n = 11)	Placebo (n = 10)	P value	Rasagiline (n = 8)	Placebo (n = 8)	P value
ETDRS letters, mean (SD)	74.9 (9.1)	72.7 (19.5)	0.74	76.8 (8.8)	77.5 (18.3)	0.92
≥65 letters	9 (82%)	7 (70%)	0.64	7 (88%)	6 (75%)	1
Gained letters, mean (SD)	61.5 (18.1)	55.3 (29.2)	0.56	68.8 (9.1)	50.8 (30.6)	0.13

ETDRS = Early Treatment Diabetic Retinopathy Study.

### Anatomical outcome

**Figure 3:**
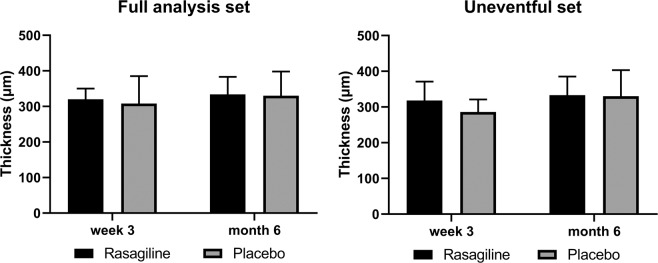
CRT change from week 3 to month 6 of the participants in the FAS and the uneventful set. No significant difference in CRT was observed from week 3 to month 6 in both groups of both sets (P > 0.05). There was no difference at either visit between the rasagiline and placebo groups (P > 0.05).

CRT was stable from week 3 to month 6, and there was no difference between the rasagiline and placebo groups for the CRT at either visit (FAS: p = 0.6 at week 3, p = 0.9 at month 6; Uneventful set: p = 0.2 at week 3, p = 0.9 at month 6, Fig. 3).

**Figure 4:**
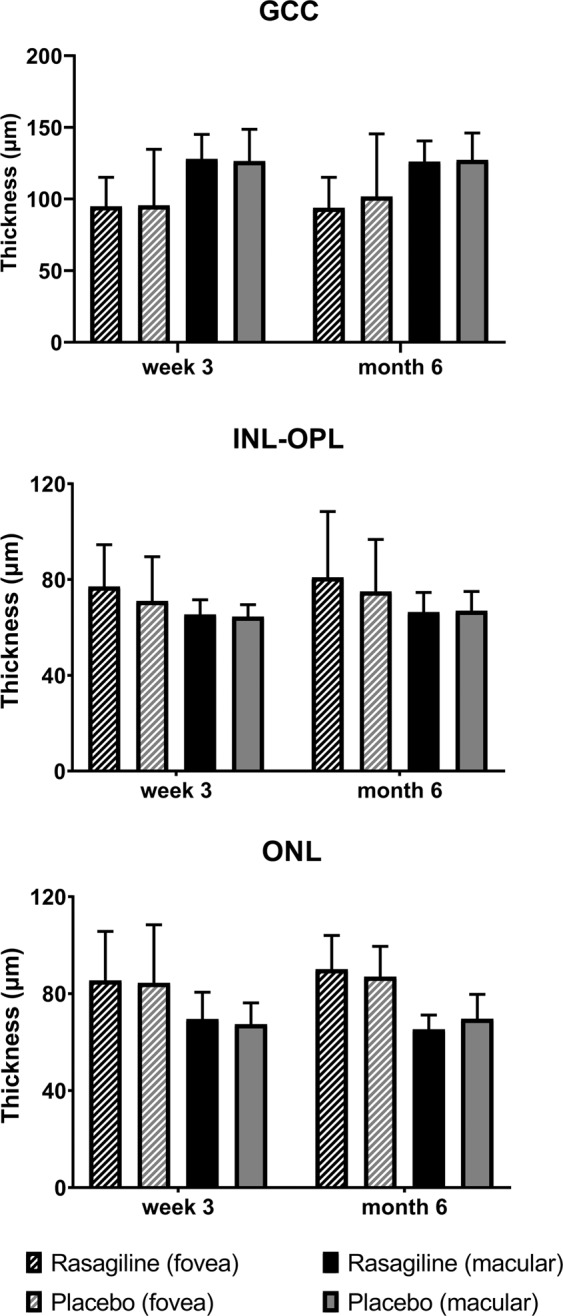
Thickness change of GCC, INL-OPL and ONL from week 3 to month 6 of the participants in the FAS. No significant thickness difference in GCC, INL-OPL and ONL was observed from week 3 to month 6 in both groups of both sets (P > 0.05). There was no difference at either visit between the rasagiline and placebo groups (P > 0.05).

Thicknesses of GCC, INL-OPL and ONL were stable from week 3 to month 6, and there was no difference between the rasagiline and placebo groups for CRT at either visit (regardless whether in the FAS or in the uneventful set (Fig. 4)).

**Figure 5:**
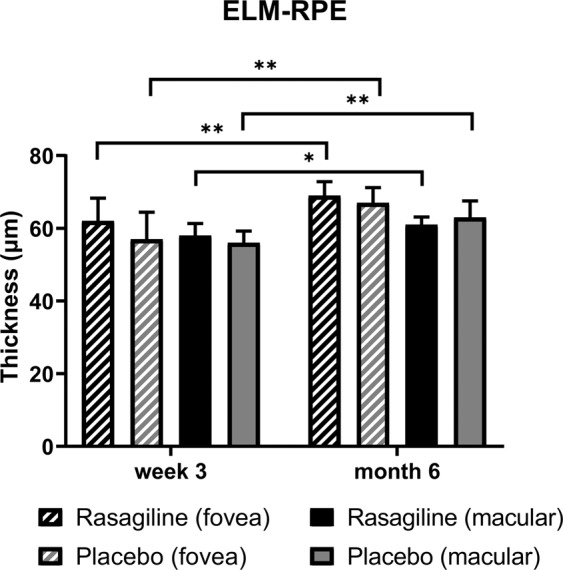
ELM-RPE thickness change from week 3 to month 6 of the participants in the FAS. ELM-RPE thickness significantly increased from week 3 to month 6 in both groups (p < 0.05). There was no difference between the rasagiline and placebo groups at either visit (P > 0.05). *p < 0.05, **p < 0.01.

ELM-RPE thickness significantly increased from week 3 to month 6, but there was no difference between the rasagiline and placebo groups at either visit, regardless of fovea (ETDRS central 1 mm subfield) or macula (ETDRS 6 mm area) (Fig. 5). Table 4 summarizes the grading result related to prc recovery. Integrity of ELM and EZ bands significantly improved in both groups without inter-group difference. The placebo group had a slightly better CIZ band integrity at week 3 (rank: 1.0 vs 1.1, p = 0.029). The foveal bulge was seen in 6 eyes at month 6 (29%, 3 eyes in each group). Simple linear regression analysis demonstrated that male gender, EZ integrity and presence of foveal bulge are positively correlated with better final BCVA. Multiple stepwise regression analysis confirmed EZ integrity and presence of bulge as factors influencing final BCVA. (Table 5)

**Table 4:** Integrity of outer retinal bands at visits 2 (week 3) & 4 (month 6) of participants in FAS.

	Rasagiline (n = 11)			Placebo (n = 10)		
	Visit 2	Visit 4	P value	Visit 2	Visit 4	P value
ELM band integrity, rank	3.8	4.8	0.002	3.8	4.8	0.001
EZ band integrity, rank	3.3	4.6	<0.001	3.0	4.4	0.001
CIZ band integrity, rank	1.0*	1.5	0.096	1.1*	1.7	0.024

The integrity was graded at the fovea in a 1-mm-diameter area on a 5-point scale as follows: (1) line not visible; (2) line disruption >500 μm; (3) line disruption >200 μm, but <500 μm; (4) line disruption <200 μm; (5) continuous line.

*Significant difference between groups.

ELM = external limiting membrane, EZ = ellipsoid zone, CIZ = cone interdigitation zone.

**Table 5:** Factors influencing final BCVA of the participants in FAS.

	Univariate analysis		Multivariate Analysis	
	β	P value	β	P value
Preoperative				
Rasagiline treatment	−0.77	0.739	—	—
Age	−0.353	0.117	—	—
Male gender	0.551	0.010	0.302	0.120
Time before presentation	−0.320	0.158	—	—
Height of macular detachment	−0.466	0.069	—	—
Baseline BCVA	0.383	0.087	—	—
Postoperative				
Additional surgery	−0.410	0.065	—	—
Recurrent detachment	−0.282	0.215	—	—
ELM integrity	0.119	0.606	—	—
EZ integrity	0.569	0.007	**0.517**	**0.008**
CIZ integrity	0.143	0.535	—	—
ERM presence	0.009	0.970	—	—
Presence of bulge	0.457	0.037	**0.387**	**0.038**

Height of macular detachment were gradable in 16 participants (8 in each group) in the FAS.

BCVA = best-corrected visual acuity, ELM = external limiting membrane, EZ = ellipsoid zone, CIZ = cone interdigitation zone, ERM = epiretinal membrane.

### Safety

A total of 8 participants (35%) reported side effects (Table 6). No serious AE was reported. There was no difference in the number of AE or side effects between the groups. Thus, rasagiline was well tolerated in patients with RRD.

**Table 6:** Side effects reported by the participants in the safety set.

	Rasagiline (n = 12)	Placebo (n = 11)	Total (n = 23)	P value
Headache	3 (25%)	2 (18%)	5 (22%)	1
Dyspepsia	0	1 (9%)	1 (4%)	—
Rhinitis	1 (8%)	0	1 (4%)	—
Joint pain	1 (8%)	0	1 (4%)	—

## Discussion

The surgical approach is currently the only means to repair RRDs and preserve or restore vision. However, visual outcomes vary, even in patients who have had successful primary surgical repair. Prc death supposedly is the ultimate cause of vision loss after a RRD. Therefore, pharmacotherapy reducing or slowing degeneration of prcs would be a potential add-on therapy enhancing functional outcomes. Several studies targeting these pathways provide evidences that a neuroprotective strategy might potentially result in prc rescue after RRD^3,4^.

Selegiline [L-deprenyl] is an earlier selective irreversible MAO-B inhibitor used in Parkinson’s disease, and showed some neuroprotective effect in retinal cells, such as lower apoptosis and higher viability in cultured RPE that led to increased survival of retinal ganglion cells after retinal damage in rats^15^. Later, rasagiline replaced selegiline in the clinic because of 15-fold higher potency. Studies demonstrated that rasagiline influences multiple pathways related to prc death, including Caspase-3 activation and Bax/Bcl-2 expression that are involved in apoptosis, while Beclin-1expression is involved in autophagy, and Caspase-1 activation and glial fibrillary acidic protein expression are involved in inflammation^7,8^.

Despite these promising results in the animal model, no pharmacotherapies have been clinically tested yet. We, therefore, carried out this clinical pilot trial to evaluate the efficacy of pharmacotherapy as an adjunct to surgical RRD repair. This study prospectively assessed rasagiline 1 mg/day treatment for 7 days in pseudophakic macula-off RRDs. We found no measurable effect of rasagiline compared to placebo. Our findings thus fell short of identifying a clinical neuroprotective effect of rasagiline at this dose on patients who had surgical repair within 96 hours (maximal time interval between vision loss and presentation plus waiting time to start of operation for surgical repair) of onset of symptoms.

Failure of rasagiline to show any neuroprotective effect in our study could be due to several reasons: First, the dose tested or the treatment duration might have been insufficient. We decided to apply this conservative regimen in the first clinical trial of rasagiline in retinal disease based on two facts: (1) the standard 1 mg dose given to Parkinson’s disease patients is well tolerated; (2) 7-day rasagiline administration was regarded as minimal duration for treatment^16^. However, with regard to the treatment dose, the two preclinical studies showing neuroprotection of rasagiline on prc used 2 μg/g BW/d in Prph2/rds mice and 15 μg/g BW/d in rd10 mice^7,8^. This would be equivalent to a daily dose of 10 mg and 75 mg for a 60-kg human. These doses are much higher than commonly used in clinic, and the second dosage would even exceed the reported tolerability range in human^17^. With respect to treatment duration, experience from the treatment of Parkinson’s disease suggests that rasagiline is limited in efficacy, but has a consistent benefit with chronic administration^18^. Hence, 7 days of treatment might not have been long enough.

Moreover, the follow-up intervals might have been unsuitably defined to evaluate pcr rescue. Differences might have been present around the 3 weeks time point. Prc death in human occurred as early as 12 h, peaked at around 2–3 days and dropped to low levels by 7 days after RRD^19^. Meanwhile, rasagiline achieves 80% MAO-B inhibition after a single standard dose and increases to 99% after 7-day administration. After treatment withdrawal, the inhibitive effects decline time-dependently and lasts for utmost 3 weeks^20^. Taken together, more benefit from neuroprotection might result from faster availability. In addition, Eigeldinger-Berthou *et al*. stated in their preclinical study that prc death was only delayed but not absent after rasagiline treatment^7^. In our study, prc evaluation through OCT images was only available 3 weeks after surgery when the SF6 gas had disappeared from the macular area. Therefore, our failure to find difference between groups from 3 weeks to 6 months after surgery might be due to bad timing, imposed by technical constraints. Novel *in vivo* evaluation would be helpful to reveal the prc change in the early postoperative phase. Ahn *et al*. demonstrated that swept-source OCT performed significantly better than SD-OCT in macular visualization in gas-filled eyes at days 1 and 3 after surgery^21^. However, their research only focused on the visualization of macular configuration, but not of the outer retinal bands. Fluorescence Lifetime imaging ophthalmoscopy (FLIO) might be a promising future option. FLIO measures lifetimes of endogenous retinal fluorophores after excitation using a picosecond pulsed blue laser light^22^. Lipofuscin is a major endogenous retinal fluorophore, and accumulates after RPE cells aging, which originates from incomplete degradation of prc outer segment^23^. Therefore, FLIO might be valuable to visualize prc death after RRD. No study has covered this topic and further research is needed.

Furthermore, other pathways might outweigh the beneficial effects attributed to rasagiline (i.e., MAO-B inhibition, effects on oxidative stress, mitochondrial dysfunction and antiapoptotic properties) in the context of RRD. In addition to prc death, RRD also causes complex cellular remodeling of neuron synapses and Müller cells, which hamper visual function and prc regeneration, respectively^24^. Furthermore, prc survival and death pathways are trigged at the same time after detachment, when some mechanism works as a scroll bar pulling prc toward survival or death, such as calpain activation. Such activation was found to be a key step in triggering prc to shift from survival to death, which peaks 7 days after detachment^25^.

The duration of macular detachment influences visual outcome greatly, because proapoptotic factors and inflammation cytokines become significantly upregulated with increasing duration of separation. Such upregulation leads to more disruption of the EZ and worse BCVA after surgery^26,27^. In this study, we only included pseudophakic macula-off RRD patients with detachment duration less than 72 hours to avoid time bias and influences of lens opacity. This could explain why our visual outcome was better than reported in other reports for patients with longer detachment duration. In our study, the mean BCVAs were 74.9 and 72.7 letters in the rasagiline and placebo group, respectively, and more than 76% patients achieved a BCVA of 65 letters or better at month 6. In a recent study, the final mean BCVA was reported to be merely 62 letters and only 61.1% of the eyes achieved 65 letters or better after a 24-month recovery after vitrectomy. Although the initial reattachment rate of 94.5% was high, their pseudophakic macula-off RRD participants had a long detachment duration of 11 days (range, 3–30 days)^28^. Of note, our regression analysis showed that there was no influence on final BCVA whether time before presentation was less than 24 hours or between 24 to 72 hours (regression coefficient: −0.320, p = 0.158). With respect to visual acuity, previous work has shown that best outcomes were achieved in patients with macular detachment of less than 7 days duration. There was no difference between patients with macular detachment of 0–3 days duration and those with central detachment of 4–7 days^1^. Our analysis suggests that there was no difference between patients with less that 24 h macular detachment and those with 2–3 days of macular involvement.

The height of macular detachment was previously reported to influence visual recovery. Lower height of macular detachment correlates with better visual outcome^29,30^. This current study observed a consistent negative association between the macular detachment height and the final BCVA, but this trend was not statistically significant (regression coefficient: −0.466, p = 0.069).

We achieved a similar SOSR of 70% in pseudophakic macula-off RRD as the rate of 63% reported in an earlier study^31^. However, the primary failure rate was 26% in our study, and was higher than the corresponding rate of 10.5% reported in previous reports on pseudophakic RRD^14^. In the current study, two PVR cases result in a PVR rate of 9%, which is rather high but still within the normal range of PVR incidence after RRD surgery cases (5–10%)^32^. Enders *et al*. studied 2457 patients with RRD and found macular detachment was not only associated with worse visual outcome but also with a higher recurrent detachment rate^33^. Therefore, we speculate that macula-off status might have contributed to our relatively high failure rate. Recurrent detachment and interventions to treat recurrent detachment have in general a worse final visual outcome than primarily successful cases. In our study, 33% of the recurrent detachment cases reached final BCVA of 70 letters or better when the detachment was managed by only one additional surgery. However, no patient achieved this level of visual acuity when more interventions were needed. This finding is in accordance with the data from Enders *et al*. that the share of good BCVA decreased from 16.9% in cases with one recurrence to 7.6% in cases with 2 recurrences and to 0% for patients that had 3 interventions^33^.

We observed significantly increased ELM-RPE thickness during our follow-up. To reveal more details of prc restoration, we graded the integrity of the ELM, EZ and CIZ, and noticed significant improvement of integrity of ELM and EZ in both the rasagiline and the placebo groups. Our finding is in line with a previous report where the prc layer demonstrated a time-dependent thickness increase after successful surgery^34^. This increase might indicate prc regeneration after detachment-induced disruption of prc outer segments, which can be visualized on OCT as dropouts of the EZ layer^35^. Taking a closer look at ELM-RPE thickness, the inner segment, ELM-EZ thickness usually achieves fully-restoration at month 6. The outer segment, EZ-RPE thickness, however, stayed thinner than that in the healthy fellow eyes after 12-month recovery^36^. The recovery of EZ-RPE thickness is the basis of the reconstitution of the foveal bulge, a sign of favourable foveal configuration^36^. We observed a foveal bulge in 29% of the eyes at month 6, in line with the study from Hasegawa *et al*., who reported a bulge in 28.6% of patients 5–8 months after a macula-off RRD^13^. Increased EZ-RPE thickness, presence of a foveal bulge and better integrity of EZ band indicate good regeneration of prc outer segments and predict better visual outcome^36,37^. Our multiple regression model showed the same result, namely that EZ integrity and presence of foveal bulge are factors predicting better BCVA.

There are many limitations to the present study. First, this clinical trial tested a very conservative regimen. This might be one major reason for our non-significant results for efficacy of rasagiline. Secondly, the sample size is relatively small, which results in low statistical power. Since this was a pilot trial, sample size calculations were not rigorous but rather pragmatic. Whitehead *et al*. suggested a pilot trial sample size per treatment arm of 20 subjects for small standardized differences (0.1–0.3) to aim for a power of 80% in a subsequent main trial^38^. In the original pilot trial protocol, we planned to enroll 20 patients in each arm. However, the trial was stopped early due to slow enrollment, resulting in a sample size of around 10 patients in each arm, which should still be appropriate to detect medium standardized differences (0.3–0.7) as suggested in above paper by Whitehead *et al*.. Thirdly, the maximum time of foveal detachment in this study was 96 hours, and we didn’t see any difference in the primary outcome. However, this doesn’t mean that one might see a neuroprotective effect in patients with longer waiting periods between foveal affection and surgical repair. Since the detachment duration is relevant for the level of prc death in RRD, the therapeutic/anti-apoptotic effect of being re-attached may have exceeded that of rasagiline in our setting. Hence, the design of the study might just have been inappropriate in this regard. Last but not least, the integrity of the outer retinal bands and the presence of a foveal bulge were manually determined, with possible bias. Despite these limitations, this study provides some prospective information on the effects of rasagiline as pharmacologic adjunct treatment for surgical RRD repair.

## Conclusion

Perioperative treatment with rasagiline 1 mg/day for 7 days did not show significant benefits for either visual or anatomical outcomes 6 months after surgery surgical repair of a macula-off RRD. EZ integrity and the presence of a foveal bulge were significant predictors of good final BCVA.

## Data Availability

The dataset generated and analysed during the current study is available from the corresponding author on reasonable request.
